# Progesterone Withdrawal-Evoked Plasticity of Neural Function in the Female Periaqueductal Grey Matter

**DOI:** 10.1155/2009/730902

**Published:** 2008-12-02

**Authors:** T. A. Lovick, A. J. Devall

**Affiliations:** Department of Physiology, University of Birmingham, Birmingham B15 2TT, UK

## Abstract

Cyclical changes in production of neuroactive steroids during the oestrous cycle induce significant changes in GABA_A_ receptor expression in female rats. In the periaqueductal grey (PAG) matter, upregulation of *α*4*β*1*δ* GABA_A_ receptors occurs as progesterone levels fall during late dioestrus (LD) or during withdrawal from an exogenous progesterone dosing regime. The new receptors are likely to be extrasynaptically located on the GABAergic interneurone population and to mediate tonic currents. Electrophysiological studies showed that when *α*4*β*1*δ* GABA_A_ receptor expression was increased, the excitability of the output neurones in the PAG increased, due to a decrease in the level of ongoing inhibitory tone from the GABAergic interneurones. The functional consequences in terms of nociceptive processing were investigated in conscious rats. Baseline tail flick latencies were similar in all rats. However, acute exposure to mild vibration stress evoked hyperalgesia in rats in LD and after progesterone withdrawal, in line with the upregulation of *α*4*β*1*δ* GABA_A_ receptor expression.

## 1. Introduction

The periaqueductal (PAG) grey matter is involved in regulating a remarkable number of bodily functions. Circuits controlling nociception, temperature regulation, micturition, vocalisation, cardiorespiratory function, and sexual behaviours are all dependent on the functional integrity of this midbrain region [1–5]. The PAG is also involved in producing emotional changes, particularly those associated with fear and defensive behaviour [6, 7], and has the ability to modulate activity in its various control circuits to orchestrate changes in the behavioural response pattern that are appropriate to an ever-changing environment [8]. In females, the PAG operates within a constantly changing hormonal milieu that results from the cyclical changes in production of neuroactive gonadal steroids during the menstrual cycle (oestrous cycle in animals). The lipophilic nature of these molecules means that they readily gain access to the brain from the circulation [9]. Here we review the results of our recent investigations into the functional consequences of changes in circulating levels of progesterone during the oestrous cycle in female rats. These experimental studies have revealed remarkable hormone-linked changes in the intrinsic excitability of the PAG circuitry that are reflected by significant changes in the behaviour of the animal.

## 2. Cyclical Changes in Progesterone Secretion in Females

In women, production of progesterone undergoes substantial changes during the menstrual cycle. Plasma levels of the steroid remain at a constant low level during the first half of the cycle. Following ovulation, secretion of progesterone by the corpus luteum increases, resulting in elevated blood plasma levels [10]. In the absence of a fertilised ovum the corpus luteum then degenerates, with an associated rapid fall in plasma progesterone production prior to menstruation.

It has long been recognised that the cyclical production of gonadal hormones during the menstrual cycle can trigger significant changes in psychological status. In up to 75% of women, the late luteal phase of the cycle, when progesterone levels decline rapidly, is associated with the development of adverse psychological symptoms; these may include irritability, mood swings, aggression, and anxiety [11]. Additionally, bodily changes such as breast tenderness and bloating may occur and responsiveness to painful stimuli becomes enhanced [12, 13]. Importantly, symptoms fail to develop in women during anovulatory cycles [14] indicating a causal relationship between changes in gonadal steroid levels and brain function. The oestrous cycle of rodents acts as a suitable model of the human menstrual cycle, and offers the opportunity to study hormone-induced plasticity of brain function within intact circuits and to relate this to a behavioural outcome. The late dioestrus (LD) phase in rats, when progesterone levels are falling naturally, correlates with the premenstrual phase in women and increased anxiety and aggression have been reported to develop during dioestrus in rats [15–17]. In rats progesterone levels also fall rapidly during proestrus following the preovulatory surge [18]. However, the dynamics of this short-lasting surge in progesterone production are not sufficient to trigger the long-lasting changes in GABAergic function that accompany LD (see below, also [19] for discussion of this point).

## 3. Neural Actions of the Progesterone Metabolite Allopregnanolone

Within the brain, progesterone produces genomic effects via neuronal nuclear-bound receptors. In addition, it can also act at the cell membrane level. These nongenomic effects are mediated not by progesterone itself but via the actions of its neuroactive metabolite 3 alpha-hydroxy-5 alpha-pregnan-20-one (allopregnanolone, ALLO). ALLO is a steroidal compound that is synthesized *de novo* within the brain from progesterone via the actions of 5*α*-reductase and 3*α*-hydroxysteroid dehydrogenase (for review, see [20]). ALLO acts at two sites on the GABA_A_ receptor. The first is an activation site that produces direct receptor activation and the second is a potentiation site at which ALLO acts as a powerful positive allosteric modulator to enhance the effects of GABA [21]. Potentiation occurs in response to low nanomolar concentrations of ALLO [22] whereas higher, micromolar concentrations, as seen for example at parturition [23], are required for direct activation of GABA_A_ receptors [24]. The levels of ALLO present in the brain are influenced by the circulating levels of progesterone and we have shown recently that ALLO from the plasma gains ready access to the PAG where it produced a decrease in neuronal excitability via a GABA_A_-mediated mechanism [25]. In other brain structures, changes in the concentration of ALLO following fluctuations in the level of circulating progesterone have been shown to trigger upregulation of GABA_A_ receptor subunit expression that leads to changes in neural excitability and behaviour [26].

## 4. Progesterone Withdrawal-Induced Plasticity of GABA Receptor Function in the PAG

GABA_A_ receptors are pentameric structures that surround a single chloride channel. Although most receptors are comprised of only 3 subunit types, the large pool of available subunits means that receptors can be comprised of many different subunit combinations [27]. The functional characteristics of individual receptor subtypes are determined by their subunit composition. There are several indications that fluctuations in the concentrations of progesterone play a major role in determining the temporal pattern of expression of certain subunits of the GABA_A_ receptor. For example, withdrawal of cultured cerebellar granule cells or cortical neurones from long-term treatment with progesterone was accompanied by upregulation of *α*4 subunit mRNA [28, 29]. Similarly, hippocampal tissue from progesterone-withdrawn rats showed upregulation of both *α*4 and *δ* subunit mRNA [26, 30]. These effects were mediated not by progesterone itself but by its neuroactive metabolite ALLO and presumably represent a response of the neurone in an effort to maintain homeostasis. Thus progesterone influences neural function directly via a genomic action at the nuclear progesterone receptor and indirectly via a nongenomic action of ALLO at the membrane-bound GABA_A_ receptor.

**Figure 1:**
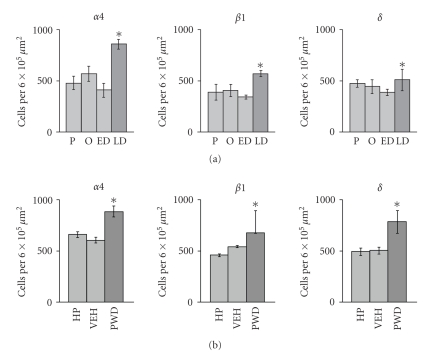
Density of *α*4, *β*1, and *δ* GABA_A_ receptor subunit-immunoreactive neurones in the PAG in (a) spontaneously cycling female rats and (b) rats that had undergone a progesterone withdrawal regime. Abbreviations: P: proestrus; O: oestrus; ED: early dioestrus; LD: late dioestrus; HP: high progesterone; VEH: vehicle treated; PWD: progesterone-withdrawn. *n* = 5 for each group, all values mean ± SEM. *P < .05, post hoc Fischer test after significant (*P* < .05) one-way ANOVA. Data redrawn from [31, 32].

**Figure 2:**
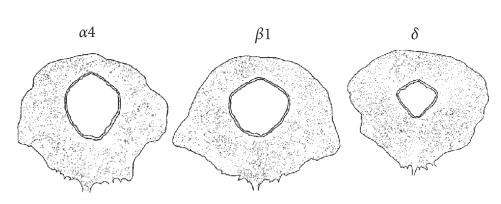
Camera lucida reconstruction showing distribution of *α*4, *β*1, and *δ* GABA_A_ receptor subunit positive neurons in representative sections taken from the mid PAG level from a rat in early dioestrus. The figure is adapted from [31].

The PAG is another brain region that shows a susceptibility to phasic changes in the ambient level of progesterone and hence ALLO. Using immunohistochemistry, we found that 24-hour withdrawal of female rats from long-term dosing with exogenous progesterone (5 mg Kg^−1^ IP twice daily for 6 days) leads to upregulation of *α*4, *β*1, and *δ* GABA_A_ receptor subunit protein in neurones in the PAG (Figure 1) [31]. Within the PAG, subunit-immunoreactive neurones were distributed throughout all subdivisions (Figure 2). However, upregulation after progesterone withdrawal was most marked in the dorsolateral column [32] where the density of GABAergic neurones, in which most *α*4*β*1*δ* receptors are expressed [31], was the greatest [33].

**Figure 3:**
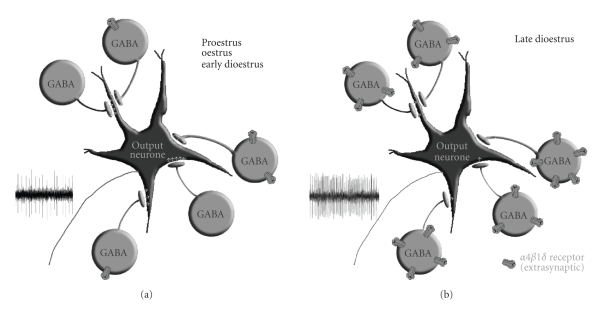
Cartoon to depict changes in GABA_A_ receptor expression in the PAG at different stages of the oestrous cycle. *α*4*β*1*δ* GABA_A_ receptors are expressed mainly by GABAergic interneurones where they are extrasynaptically located and mediate tonic currents. The excitability of output neurons from the PAG is limited by spontaneous activity in GABAergic interneurones. (a) When expression of *α*4*β*1*δ* GABA_A_ receptors is low during proestrus (Pro), oestrus (O), and early dioestrus (ED), high levels of activity in the interneurone population limit the excitability of the output neurons. (b) When progesterone levels fall during late dioestrus (LD), increased expression of *α*4*β*1*δ* receptors leads to an increase in tonic current carried by GABAergic cells, thus limiting their on-going activity. The output neurones therefore become intrinsically more excitable, and their threshold for activation is lowered. The figure is adapted from [38].

We have been able to translate the findings obtained using an exogenous dosing regime to the natural fluctuations in the hormone that occur during the oestrous cycle. During LD, when plasma levels of progesterone are falling, a parallel upregulation of *α*4, *β*1, and *δ* GABA_A_ receptor subunit protein occurred within the PAG [34]. This suggests that new GABA_A_ receptors with the *α*4*β*1*δ* subunit configuration had been formed in response to the falling steroid levels in the brain. GABA_A_ receptors containing *δ* subunits are likely to be extrasynaptically located [35] and those with the *α*4*β*1*δ* configuration are characterised by an extremely low EC_50_ for GABA [36]. The *α*4*β*1*δ* GABA_A_ receptors in the PAG should therefore be activated by the level of GABA present in the extracellular fluid and be responsible for mediating tonic currents [35, 36]. Expression of *α*4, *β*1, and *δ* subunit protein was confined predominantly to the GABAergic neuronal population in the PAG [32]. These two factors, that is, sensitivity to GABA and cellular location, may provide an important key to the functional consequences of progesterone withdrawal-induced receptor plasticity. In functional terms, increased expression of *α*4*β*1*δ* receptors on GABAergic neurones would be expected to lead to a reduction in the level of their activity, thus disinhibiting the output neurones within the PAG by reducing the level of ongoing GABAergic tone (Figure 3). Hence, one would expect to see an increase in the excitability of the various neural control systems located within the PAG. The PAG is organised functionally in terms of a number of longitudinal columns that integrate and control diverse aspects of its function [37]. The oestrous cycle-linked upregulation of *α*4, *β*1, and *δ* GABA_A_ receptor subunit protein was seen in all regions of the PAG with little evidence of somatotopic localisation. Hence, the functional consequences of receptor plasticity are likely to be widespread and not confined to any one aspect of PAG function.

## 5. Oestrous Cycle-Linked Changes in Neural Excitability of the Female PAG

The excitability of the neural circuits of the PAG is normally regulated by tonic activity of GABAergic neurones. At the neuronal level, the presence of ongoing GABAergic inhibition within the PAG was revealed by the increase in firing rate of output neurones in the presence of a GABA_A_ antagonist [19]. In the conscious rat, the functional importance of the tonically active GABAergic control system is manifested by the dramatic behavioural changes elicited by microinjection of GABA_A_ antagonists into this region [39–42]. To date, most of these studies have been restricted to male animals. However, given the plasticity of the GABAergic control system in the PAG in females, changes in the functional excitability of the neural circuitry might be expected to occur at different stages of the oestrous cycle. Indeed, our electrophysiological studies have shown that GABA tone in the PAG in females is reduced during LD [19] and also in oestrus, although the latter effect is unlikely to be related to plasticity of *α*4*β*1*δ* receptor expression (for a discussion on this point see [19]). Changes in the intrinsic level of GABAergic tone in the PAG have the potential to impact significantly on the wide range of the behaviours that are controlled by this region. Indeed, even in the anaesthetised preparation, we were able to show changes in responsiveness of single neurones in the PAG to the panicogenic and pronociceptive CCK_2_ receptor agonist pentagastrin at different stages of the cycle [19].

## 6. Behavioural Consequences of Steroid Hormone Withdrawal-Evoked Changes in PAG Function

The PAG is a source of multiple descending control pathways to the spinal cord that exert both inhibitory and facilitatory influences on the spinal processing of nociceptive information [43]. Both pro- and antinociceptive control systems are thought to be tonically active under normal conditions but the balance between them is in a state of constant dynamic flux [44, 45]. We have recently begun to investigate the behavioural consequences of steroid hormone-evoked changes in the excitability of the circuitry in the conscious animal, focussing on changes in responsiveness to painful stimuli as well as on indices of anxiety.

The tail flick latency (TFL) in response to radiant heat applied to the underside of the tail elicits a withdrawal reflex that is a commonly used index of sensitivity to acute cutaneous pain in conscious rats [46]. We compared TFLs in rats that had undergone a progesterone withdrawal regime (5 mg Kg^−1^ IP twice daily for 6 days followed by 24-hour withdrawal) with those obtained from rats in proestrus (Pro) and LD. These two stages of the oestrous cycle were chosen as being representative of low and high expressions of *α*4*β*1*δ* GABA_A_ receptors in the PAG (see Figure 1). Since changing hormone levels might also induce changes in stress or anxiety levels in rats used for nociception testing, which could potentially influence their perception of pain [46], we also observed the behaviour of animals in a 1 m × 1 m open field arena to assess intrinsic anxiety levels [47].

**Figure 4:**
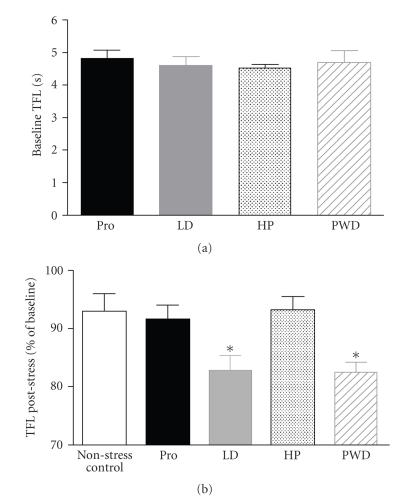
(a) Baseline tail flick latency in spontaneously cycling female Wistar rats in proestrus (Pro) and late dioestrus (LD) and in rats undergoing progesterone treatment (HP) or withdrawal from an exogenous progesterone dosing regime (PWD). Data show mean values of three readings taken at 5-minute intervals. All values mean ± SEM. Pro: *n* = 13, LD: *n* = 12, HP: *n* = 8, PWD: *n* = 7. (b) Change in tail flick latency (TFL) following 5-minute exposure to nonnoxious vibration stress in normally cycling and progesterone-withdrawn female Wistar rats. Data represent mean values obtained during 10 minutes immediately poststress as a percentage of the mean baseline level measured during 10 minutes prior to the stress. A control group comprising rats in proestrus and late dieostrus received no vibration stress. All values mean ± SEM; no stress (control): *n* = 12, Pro: *n* = 13, LD: *n* = 12, HP: *n* = 8, PWD: *n* = 7. *P < .05, post hoc Dunnett’s test in comparison to control group after significant (*P* < .05) one-way ANOVA.

Experiments involving nociceptive testing were carried out under the UK Animals (Scientific Procedures) Act 1986 and conformed with the Guide for the Care and Use of Laboratory Animals published by the US National Institutes of Health (NIH Publication no. 85–23, revised 1985). Female Wistar rats were habituated to a restraining tube. TFLs were measured at 5-minute intervals over a 15-minute period, that is 3 tail flick tests, to obtain a basal value for TFL. There was no difference in basal TFLs between any of the different groups of spontaneously cycling rats or the progesterone-treated animals (Figure 4(a)). Similarly, there was no difference in any of the behavioural indices measured in the open field (Table 1). We next investigated interactions between anxiety and nociception. The rats were subjected to mild nonnoxious stress by vibrating the restraining tube with the rat inside it for 5 minutes at 4 Hz [48]. During the vibration stress, the rats showed signs of anxiety that included micturition, defaecation, and vocalisation. Immediately following the vibration stress, tail flick testing was resumed. Rats that were undergoing progesterone-withdrawal had developed a poststress hyperalgesia that was manifested as a significant decrease in tail flick latency that persisted for 10 minutes (Figure 4(b)). Rats maintained on the progesterone dosing schedule failed to develop hyperalgesia (Figure 4(b)). Spontaneously cycling rats in Pro (low *α*4*β*1*δ* GABA_A_ receptor expression in the PAG) were compared with rats in LD (high *α*4*β*1*δ* GABA_A_ receptor expression in the PAG) using the same protocol. Rats in LD displayed a significant hyperalgesia following the vibration stress, whereas rats in Pro showed no change in TFL (Figure 4(b)).

These experiments failed to show any change in baseline thermal nociception in female Wistar rats at different stages of the oestrous cycle. In contrast, other investigators have been able to detect oestrous cycle-linked differences in sensitivity to pain [49–53]. However, the reports of differences in nociception with respect to cycle stage were equivocal. At best, it seems that cycle stage may influence responsiveness to pain in rats but only in some strains and under specific experimental conditions. In terms of PAG function, this suggests either that there is very little oestrous cycle-linked change in tonic descending control of spinal nociceptive processing or, alternatively, that the activity in the control systems is altered during the cycle but in such a way that there is little change in the net balance of control at the spinal cord level.

In line with the lack of oestrous cycle effect on basal pain sensitivity, we were also unable to detect any differences in basal anxiety levels using the open field test. This finding is supported by previous studies [54, 55] but not by the work of Frye et al. [56] who reported an increase in anxiety-related behaviour in a brightly lit open field during the dark phase of the day in rats in dioestrus. However, this study may not be directly comparable to the present work, since testing in bright light, as opposed to the relatively subdued lighting (60 lux) used in the present study, would be inherently more stressful to the rats [57] by compounding the anxiogenic effects of bright light and exposure to a novel environment.

Interestingly, in the present study oestrous cycle-related differences in responsiveness to pain became apparent in the setting of a mild stress that increased anxiety levels. Moreover, the oestrous cycle-linked hyperalgesia appeared only during LD, when *α*4*β*1*δ* receptor expression in the PAG would be upregulated. Rats undergoing withdrawal from progesterone, in which *α*4*β*1*δ* receptor expression in the PAG would also be upregulated, showed a hyperalgesia, indicating that the effect seen in the spontaneously cycling animals was likely to be hormone-linked. During the oestrous cycle, levels of a number of gonadal hormones change [18]. Oestradiol in particular has been shown to affect GABAergic function [58]. However, at the time when *α*4*β*1*δ* GABA_A_ receptor expression increase during LD, plasma oestradiol levels are low and stable, suggesting that changes in progesterone rather than oestrogen are responsible for the changes in PAG function during LD. The results of our most recent work indicate that the PAG may be involved in mediating the oestrous cycle-linked hyperalgesia in the setting of mild anxiety. We have found that exposure to the vibration stress regime elicits differential expression of the immediate early gene c-*fos* in the ventrolateral PAG in rats in Pro and LD (Lovick and Devall, unpublished work). The ventrolateral PAG contains neurons that are a source of descending facilitation of spinal nociceptive inputs [59, 60]. Thus steroid hormone-linked changes in the excitability of descending control systems from the PAG may alter the level of descending control over spinal nociceptive processing and contribute to the hyperalgesia that develops in LD or during progesterone withdrawal.

Recent imaging studies in humans have also implicated the PAG in anxiety-induced hyperalgesia. Anticipatory anxiety in expectation of receiving a painful cutaneous thermal stimulus led not only to a heightened pain experience when the stimulus was delivered, that is hyperalgesia, but was also associated with activation of the PAG [61]. In women, increased responsiveness to noxious stimulation has been reported consistently during the luteal phase of menstrual cycle [12, 13]. In many women, anxiety levels are raised during the late luteal phase [11]. In any experimental scenario involving pain testing in human subjects, a degree of anxiety or apprehension is almost inevitable. Thus it is possible that the reported menstrual cycle-related differences in pain sensitivity in women may to a large extent be secondary to changes in anxiety levels rather than a primary response to changing hormone levels.

In female rats, falling progesterone levels can produce remarkable changes in the functional characteristics of neurones in the PAG, which may underlie certain oestrous cycle-linked changes in behaviour. These findings have implications for the design and interpretation of behavioural studies in female rodents in which the stage of the oestrous cycle may be a significant confounding influence. It is also possible that such hormone-linked changes may underlie the development of menstrual cycle-related disorders in susceptible women.

## Figures and Tables

**Table 1:** Behavioural indices in open field test for rats in proestrus, late dioestrus, and after progesterone withdrawal. All values mean ± SEM. Late dioestrus (LD, *n* = 15), proestrus (Pro, *n* = 22), high progesterone (HP, *n* = 7).

Measure	Group		
	LD	Pro	HP
Total distance travelled (cm)	3827 ± 228	3737 ± 205	4328 ± 208
Average speed (cm s^−1^)	12.85 ± 0.76	12.91 ± 0.70	14.45 ± 0.70
Time in central zone (%)	2.56 ± 0.51	1.96 ± 0.25	2.82 ± 0.56
Time in middle zone (%)	10.00 ± 1.98	7.74 ± 1.42	6.75 ± 0.94
Time in outer zone (%)	86.60 ± 2.24	89.09 ± 1.73	90.85 ± 1.43
Number of central zone re-entries	4.25 ± 0.64	4.10 ± 0.62	3.57 ± 0.57
Time rearing (%)	18.53 ± 2.35	19.13 ± 2.01	14.13 ± 1.26
Time freezing (%)	0.30 ± 0.30	0.54 ± 0.44	n/a
Time grooming (%)	7.68 ± 1.51	6.48 ± 1.48	4.93 ± 1.38
